# Supplementary figures and images for: Increasing salinity of fibrinogen solvent generates stable fibrin hydrogels for cell delivery or tissue engineering
Source: PLoS One. 2021 May 19;16(5):e0239242. doi: 10.1371/journal.pone.0239242 (PMC8133424; doi:10.1371/journal.pone.0239242)

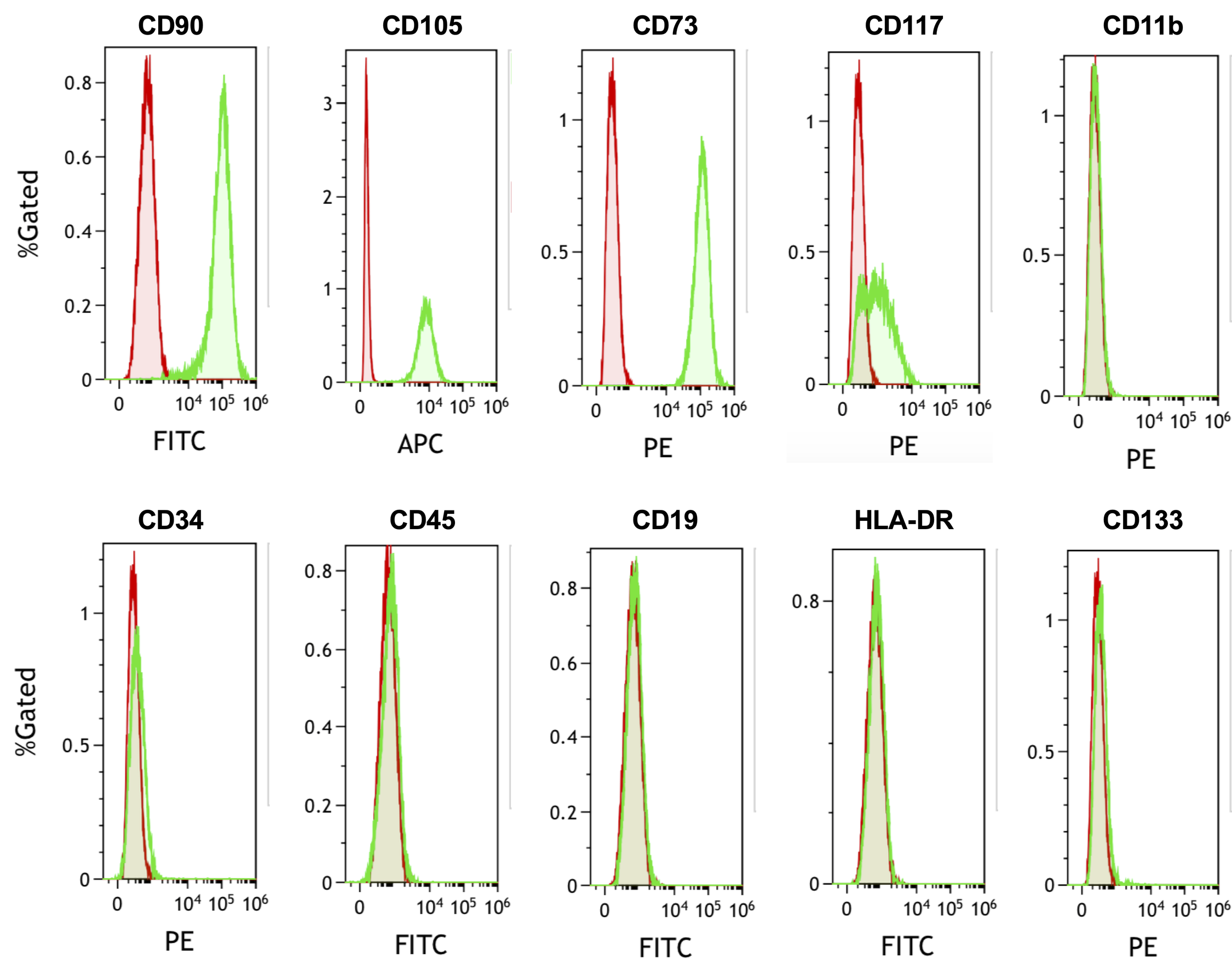

Supplement: S1 Fig — (TIF) [file pone.0239242.s001.tif]
