# Supplementary material for: Increasing salinity of fibrinogen solvent generates stable fibrin hydrogels for cell delivery or tissue engineering
Source: PLoS One. 2021 May 19;16(5):e0239242. doi: 10.1371/journal.pone.0239242 (PMC8133424; doi:10.1371/journal.pone.0239242)
Supplement: S1 File — (DOCX) [file pone.0239242.s002.docx]

**Supplementary Information**


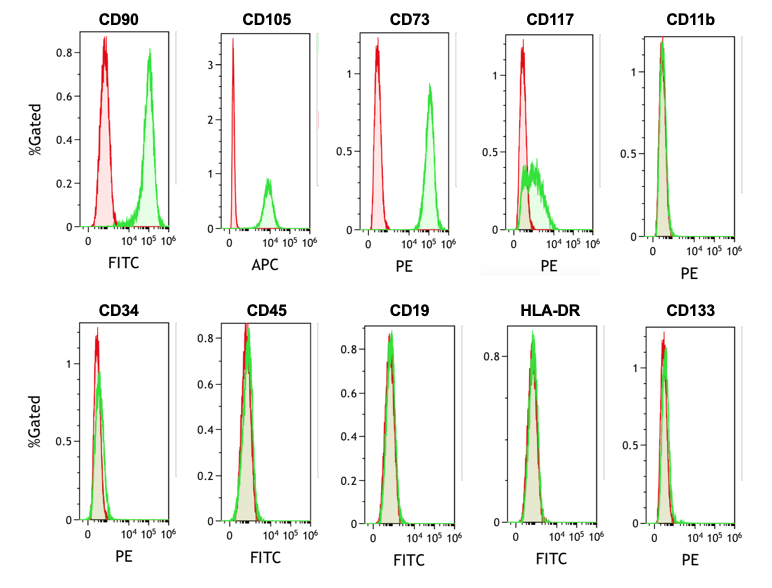


**Supplementary Methods: Flow Cytometry**

Human amniotic fluid cells (AFCs) were collected from amniotic fluid as previously described (14). Amniotic fluid was obtained from therapeutic amnioreduction procedures at Children’s Hospital Colorado.

Cells were analyzed for mesenchymal stem markers via flow cytometry. 80% confluent AFC (P4) were dissociated and labeled with CD11b (BD Biosciences, 561001), CD19 (BD Biosciences, 560994), CD34 (BD Biosciences, 560941), CD45 (BD Biosciences, 560976), CD73 (BD Biosciences, 561014), CD90 (BD Biosciences, 5555595), CD105 (BD Biosciences, 562408), CD117 (ThermoFisher, 12-1178-42), CD133 (Biolegend, 372803), and HLA-DR (BD Biosciences, 560944) for 30 minutes in 1% FBS in PBS. Cells were then washed with 1% FBS in PBS and analyzed using a Gallios 561 (Beckman Coulter) with assistance from the University of Colorado Cancer Center Flow Cytometry Shared Resource.
